# A Multipath Routing Protocol Based on Clustering and Ant Colony Optimization for Wireless Sensor Networks

**DOI:** 10.3390/s100504521

**Published:** 2010-05-04

**Authors:** Jing Yang, Mai Xu, Wei Zhao, Baoguo Xu

**Affiliations:** 1 School of Communication and Control Engineering, Jinangnan University, Wuxi, 214122, China; E-Mail: xbg@jiangnan.edu.cn; 2 College of Electrical Engineering, Guizhou University, Guiyang, 550003, China; 3 Department of Electrical and Electronic Engineering, Imperial College London, London, UK; E-Mail: maixu06@imperial.ac.uk; 4 Department of Electrical Engineering, Tsinghua University, Beijing, 100084, China; E-Mail: Zhaowei@mail.tsinghua.edu.cn

**Keywords:** wireless sensor networks (WSNs), clustering, multipath, ant colony optimization (ACO)

## Abstract

For monitoring burst events in a kind of reactive wireless sensor networks (WSNs), a multipath routing protocol (MRP) based on dynamic clustering and ant colony optimization (ACO) is proposed. Such an approach can maximize the network lifetime and reduce the energy consumption. An important attribute of WSNs is their limited power supply, and therefore some metrics (such as energy consumption of communication among nodes, residual energy, path length) were considered as very important criteria while designing routing in the MRP. Firstly, a cluster head (CH) is selected among nodes located in the event area according to some parameters, such as residual energy. Secondly, an improved ACO algorithm is applied in the search for multiple paths between the CH and sink node. Finally, the CH dynamically chooses a route to transmit data with a probability that depends on many path metrics, such as energy consumption. The simulation results show that MRP can prolong the network lifetime, as well as balance of energy consumption among nodes and reduce the average energy consumption effectively.

## 1. Introduction

The wireless sensor networks (WSNs) technology have been widely applied in military, industry, agriculture and many other areas [1,2]. In the WSNs, a lot of nodes operate on limited batteries, making energy resources the major bottleneck. Therefore, an economical and frugal management of energy is essential for improving energy efficiency. Because energy consumption due to communication is the major part of the energy consumption in WSNs [3], a high performance routing protocol is often a key requirement in WSNs systems. The design of routing protocols in WSNs is very challenging due to their inherent characteristics of large scale, no global identification, dynamic topology, and very limited power, memory, and computational capacities for each sensor. Currently, many energy-efficient routing algorithms have been studied with the aim of saving energy [4–6].

The existing routing protocols in WSNs can be categorized into flat routing protocols and hierarchical routing protocols, or single-path routing protocols and multipath routing protocols [7]. Recent research on WSNs routing protocols has proven that clustering and multipath are needed to improve energy efficiency and load balancing.

When designing multipath routing algorithms, many parameters (e.g., path length and energy consumption of communication) also need be considered. The optimization of network parameters for WSNs routing processes might be considered as a combinatorial optimization problem. Our proposed approach benefits from the success of ant colony optimization (ACO) [8] in solving the problem. The ACO algorithm is a heuristic algorithm introduced by Dorigo and his collaborators for solving some combinatorial optimization problems [9], such as traveling salesman problem (TSP) [10]. The ACO algorithm has some characteristics, such as distributed computing, self organization and positive feedback, suited for searching routing in modern communication networks.

However, few of the existing works have considered the integration of clustering, multipath and ACO to maximize the network lifetime and achieve load balancing in WSNs. Motivated by the advantages of clustering, multipath and ACO, this paper proposes a multipath routing protocol (MRP) based on dynamic clustering and ACO for reactive WSNs. The main objective of our work is to maximize network lifetime and at the same time achieve load balancing. The main contributions of this paper are listed:

1. A novel distributed algorithm based on some parameters (such as signal strength, residual energy of node) is designed to form clusters among the nodes located in the event area.
2. An extended ACO algorithm based on many metrics (such as residual energy, path length, energy consumption of communication) is applied to search for the multiple paths between the cluster head (CH) and sink node.
3. A load balancing function is further proposed to distribute the traffic over discovered multiple paths.

The rest of this paper is organized as follows. Section 2 introduces some related routing algorithms. In section 3 we propose the system model and the motivation of our work. The details of the MRP algorithm are described in section 4. The performance evaluation of our scheme as well as a comparison with the previous typical routing algorithms is presented in section 5. Section 6 draws the conclusions.

## 2. Related Work

WSNs are a kind of decentralized network of autonomous nodes that collect and process information, and send the information to a sink node over wireless links. Limited energy nodes are not taken into account in the traditional routing protocols, which has significant impact on the overall energy dissipation. Therefore, new routing protocols need be designed for WSNs.

### 2.1. Hierarchical Routing

Hierarchical (clustering) technology is particularly promising and has received much attention in the research community. In a hierarchical network, the data gathered by sensor nodes is transmitted to CHs. The sensed data from nodes within one cluster usually exhibit high correlation, and therefore, a CH can aggregate data to remove redundancy and only send one packet to the sink.

In the last few years, many hierarchical routing algorithms are proposed for WSNs. One pioneering work in the literature is LEACH (Low-Energy Adaptive Clustering Hierarchy) [11]. LEACH is an application-specific data dissemination protocol that uses clustering to prolong the network lifetime. However, the assumption that all nodes are capable of communicating with any node in the field does not allow the network to be scalable, and LEACH does not guarantee good distribution of CHs. To improve LEACH performance, Lindsey *et al.* introduced chain into clustering (power-efficient gathering in sensor information systems, PEGASIS) [12]. In this work, all nodes are connected in a chain and communicate only with the nearest neighbor. Nodes take turns to be the CH and send aggregation data to the sink. Although PEGASIS outperforms LEACH in network lifetime, it assumes that all nodes have a global knowledge of the network. Thus, PEGASIS may not be efficient with closely deployed nodes in a specific area. In [13], the authors designed an ant-based algorithm (T-ANT) to cluster and achieved a uniform distribution of CHs in the network.

### 2.2. Multipath Routing

Multipath routing uses multiple paths to transmit data, which can achieve both load balancing and fault tolerance. There are two different multipath routings between the source node and the sink node. One is disjoint multipath routing [14], where the alternative paths do not intersect with each other. The other is braided multipath routing, where there are typically no completely disjoint paths [15–16].

In [14], Ganesan *et al*. presented a disjoint multipath routing based on local information, which is a distributed algorithm and can achieve load balancing. This algorithm uses a primary route to transmit data. Only when the primary route fails, the alternative route can be used. However, this algorithm is not attractive for the network lifetime.

In [15], a meshed multipath routing with efficient strategy has been described. Such an algorithm can achieve a better throughput than the traditional multipath algorithms. However, this approach requires nodes to be equipped with GPS (Global Positioning System), which increases the cost of the node.

In [16], Okdem *et al.* introduced a multipath routing algorithm based on Ant Colony Optimization (ACO), which uses a class of agent-like ants to develop multiple reliable routes between the source and sink. It is very effective in dealing with the failure of links and searching for the routes. Due to the large number of nodes, the number of ants is quite large so that it may lead to much higher traffic in the network than other algorithms.

### 2.3. Ant Routing

As an effective distributed approach, the ACO algorithms have been introduced to the design of routing protocol and have received many achievements [17–25].

The ACO algorithm was first used in traditional networks [17]. ARA [18] was the first algorithm used in mobile ad hoc networks (MANETs), which exploits the pheromone laying behavior of ants to search for routing. The above two algorithms are however not suitable for WSNs. In [19], Liu *et al.* used an improved ACO algorithm (PACO) to search for multipaths between source nodes and the sink node in MANETs. Although the PACO improves the efficiency of data transmission, the number of ants required to search for routing is great, resulting in great energy consumption at the start-up stage. Moreover, the PACO only uses the length of path as metric without considering the current energy of nodes: these discovered paths may contain the low energy nodes, which will shorten the working time of the paths.

Recently, routing protocols based on ACO for WSNs have been the focus of many studies [20–25]. In [20], Zhang *et al.* studied three distinct Ant-based algorithms for WSNs. However, the algorithms only focus on the building of an initial pheromone distribution, and thus, the algorithms are only good at system start-up phase. In [21], Camilo *et al.* presented a new WSNs routing algorithm based on ACO, which can minimize communication load and save energy. Nevertheless, the algorithm does not consider the feature of data correlation, the energy consumption of communication is huge when a lot of sources exist in the network. In [22], Liu *et al.* introduced a routing strategy on the basis of ant algorithm for WSNs, using deflection angle, energy and distance as routing factors to help the ant to search for routing. The convergence rate of the algorithm is good. However, the algorithm did not utilize the redundancy of data, and thus the algorithm has the same disadvantage as [21]. A reinforcement learning scheme is proposed in [23], which reduces the energy consumption and shortens the time delay. The algorithm, however, only uses the distance as metric, so it cannot protect the minimum energy node, and therefore, it may shorten the network lifetime. In [24], Tu *et al.* constructed a chain by means of an ant colony algorithm that connects all the nodes in the networks. Although the algorithm can find suboptimal routing for mobile agents, the time delay of the algorithm is long, and the cost of reconstructing routing is also high. In [25], Ren *et al.* proposed a multipath routing based on ant colony system, which extends the network lifetime. Although the algorithm balances the energy consumption among nodes by multipath, it does not take into consideration the influence of the minimum energy node on multiple paths. In [26], Okdem *et al.* presented an ACO-based multipath routing, which provides good energy efficiency. However, the algorithm belongs to a kind of flat routing, therefore, its scalability is not good.

Although these algorithms presented above have some advantages, there still exist some shortcomings that prevent their application in large scale WSNs. To overcome the disadvantages of conventional ant-based routing algorithms, we propose an improved protocol by integrating the advantages of hierarchical routing, multipath routing and ACO.

## 3. System Model and Problem Statement

### 3.1. System Model

#### Network Model

A WSN consists of a large number of sensors, and wireless links representing direct communication between the sensors within the radio range. A WSN is modeled as an undirected graph G(*V,E,W*), where *V* = {*v*_1_, *v*_2_,…,*v_n_*} is the set of all the nodes. Each *v_n_* has its maximum communication radio range with radius *R. E* is the set of all bidirectional wireless links *(i, j)* (*i, j* ∈ *V*). A link *(i,j)*, denoted by *e*(*i, j*) ∈ *E*, exists between *v_i_* and *v_j_* if *d*(*v_i_*,*v_j_*) ≤ R. It indicates that node *v_i_* and node *v_j_* can directly communicate with each other. *d(v_i_,v_j_)* is the distance between node *v_i_* and node *v_j_*. W is the weight set of all directed links *(i,j)*. The weight *e_ij_* of link *(i,j)* is the energy consumption of communication between *i* and *j*. Let *S_e_* = {*v_1_*,*v_2_*,…,*v_l_*} be the set of all nodes in the event area. Note that *S_e_* is the subset of *V* (*S_e_*⊆*V*). Let *N_i_* denote the set of neighbors of node *i* and *N_i_* = {*j*|*d_ij_≤R, j* ∈ *V*}.

In this paper, the following assumptions are adopted:

- N sensor nodes are uniformly distributed within a square field. Each sensor nodes has a unique ID. Sensor nodes in the event area are grouped into clusters.
- All sensor nodes keep static or less movement after being deployed.
- The energy of the sensor nodes cannot be recharged.
- Sensor nodes are location-unaware, *i.e.*, a sensor node need not rely on the expensive devices, such as Global Positioning System (GPS), to receive the position information for finding the shortest path routing to the sink.
- Communication is symmetric. Nodes can estimate distance based on the signal strength of each other, and at the same time the radio power can be controlled.
- We assume ideal MAC layer conditions, that is, perfect transmission of data on a node-to-node link.

#### Radio Model

In order to evaluate the energy dissipation between node *i* and node *j*, we use the radio model used in [11,27]. The energy costs of transmitting and receiving a *k* bit data packet between node *i* and node *j* with distance *d* are denoted by *E_T_(i,j)* and *E_R_(i,j)* which may be computed by:

(1) $$E_{T}(i,j)=k\left(E_{\mathrm{elec}}+E_{\mathrm{amp}}\times d^{\gamma }\right)$$

(2) $$E_{R}(i,j)=kE_{\mathrm{elec}}$$

where *E_elec_* and *E_amp_* are the energy dissipation of per bit for transmitter or receiver, and the transmit amplifier, respectively; *γ* ∈ {2, 4} can be seen as the path loss exponent.

### 3.2. Problem Statement

**Figure 1.**
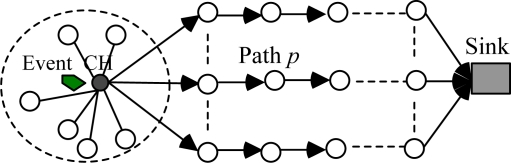
Data Transmission Model in MRP.

The MRP algorithm uses the ACO algorithm to search for multiple paths after cluster formation. The process of ants moving will result in multiple paths forming between CH and sink. After multiple paths are formed, data will be transferred along the multiple paths. The model of data transmission in MRP is shown in Figure 1.

From Figure 1, we conclude that MRP can maximize the network lifetime in two ways. One is to reduce the transmitted data by clustering, which can use data aggregation to reduce energy consumption. The other is to use multiple paths to achieve load balancing (*i.e.*, it can avoid frequently using the path in which the minimum energy node is located).

Based on the above introduction, we can describe the objective of MRP as follows: maximizing the network lifetime (*T_net_*), while minimizing the energy consumption between the CH and sink, which can be formulated as follows:

(3) $$\text{max}\;T_{\mathrm{net}},\;\text{min}\sum_{\left[i,j\in V,(i,j)\in E\right]}e_{\mathrm{ij}}$$

*Theorem 1:* Network lifetime is associated with residual energy of node, the energy consumption and the number of hops in path *p*.

In order to prove the theorem, we use a simplified model of energy consumption, in which energy consumption is the same in each node.

*Prove: N* is the set of discovered paths between the CH and sink. The energy consumption of path *p* (*p* ∈ *N*) is the sum of the energy expended at each sensor node along the path. If (*n_1_*,*n_2_*,…,*n_m_*) denotes the sequence of nodes along path *p*, the total energy consumption *E(p)* is given by

(4) $$E(p)=\sum_{k=1}^{m-1}\left(E_{r}+E_{\mathrm{cpu}}+E_{t}\right)=\left(E_{r}+E_{\mathrm{cpu}}+E_{t}\right)\times (m-1)$$

where *E_r_* and *E_t_* represent the energy consumption of receiving or transmitting *L*-bit data, respectively. *E_cpu_* is the energy consumption used in these jobs, such as computation, sensing events, *etc*.

From [3], we have

(5) $$E_{\mathrm{cpu}}<<E_{t}+E_{r}$$

Therefore, we have

(6) $$E(p)=\left(E_{t}+E_{r}\right)\times (m-1)$$

In (6), because the value of (m−1) is equal to the number of hops between the CH and sink, we have

(7) $$E(p)=\left(E_{r}+E_{t}\right)\times h_{\mathrm{CH}}(p)$$

where *h*_CH_(p) is the number of hops in path *p*. Thus, *E*_r_ + *E*_t_ can be given by

(8) $$E_{r}+E_{t}=E(p)/h_{\mathrm{CH}}(p)$$

Since the working time *T(p)* of path *p* is partly determined by the minimum energy node in path *p*, *T(p)* is given by

(9) $$T(p)=E_{\text{min}}(p)/\left(E_{r}+E_{t}\right)$$

where *E*_min_(*p*) is the current energy of the minimum energy node in path *p*.

Using (8) and (9), we have

(10) $$T(p)=E_{\text{min}}(p)\times h_{\mathrm{CH}}(p)/E(p)$$

We define the network lifetime *T*_net_ as the time when the first node in the network runs out of energy. Then, *T*_net_ is given by

(11) $$T_{\mathrm{net}}=\underset{p\in N}{\text{min}}T(p)=\underset{p\in N}{\text{min}}E_{\text{min}}(p)\times h_{\mathrm{CH}}(p)/E(p)$$

Therefore, *T*_net_ is associated with the residual energy of the minimum energy node, energy consumption in a path and hop distance to sink.

According to (3) and (11) it is obvious that maximizing the network lifetime *T*_net_ is equivalent to maximizing the minimum *T(p)*. We have

(12) $$\text{maximize}\;\underset{p\in N}{\text{min}}T(p)$$

From (12), we infer that MRP needs to prolong working time of the minimum energy node and reduce the energy consumption in a path in order to maximize the network lifetime.

## 4. Description of MRP

MRP is divided into three phases: cluster formation, multipath construction and data transmission. The first phase is executed when an event happens. Its objective is to realize dynamic clustering. In the second phase, the CH use ACO to search for multiple paths. The last phase dynamically chooses one path to transmit data according to an evaluation function.

To start the operation of the routing scheme, nodes having information for the sink initialize the routing task by transmitting an ADV message to neighbor nodes. Each node then broadcasts the ADV message to its neighbor nodes, and so on. At the end of the initiation stage of the network, each node constructs a table containing neighbor information, as shown in Table 1.

The information in Table 1 will be used to help an ant search for routing. ID indicates the identification number of a node. *τ_ci_* is the pheromone value on link *(c,i)*, which represents the local link situation and quality. At the beginning, the pheromone in the network is a constant; then, it varies with ant routing. *D_is_* is the distance between node *i* and sink, and it may be estimated by the received signal strength (*RSS*) [28]. *Hop count* is the number of hops from a node to sink. *Tag* indicates the instance of being visited by an ant. *Tag* = 1 indicates that the current node has been visited by an ant, otherwise, *Tag* = 0.

### 4.1. Phase I: Cluster Formation

The conventional hierarchical routing algorithms [11,12,27] do not fit for monitoring burst events in reactive WSNs. For example, though there is no event happening, each node will still have to flood control packets to periodically reconstruct clusters. Clustering is not related to an event, *i.e.*, nodes sensing the event locate in different clusters, which will reduce the data aggregation efficiency. If the clusters are reconstructed, an event may happen, and result in the event not being detected.

In order to overcome the disadvantages [11,12,27], MRP adopts a dynamic clustering algorithm, *i.e.*, nodes having information about an event taking place nearby will join clustering. The clustering algorithm obeys the rules as follows:

- Nodes located in the event area can sense the distance to the event according to *RSS*.
- Nodes know the residual energy of neighbor nodes in the event area.
- If *RSS_i_* ≥ Threshold Value [29], node *i* locates in the event area. (*RSS_i_* is the received signal strength of node *i*)

*Theorem 2:* When a CH locates in the center of the event area, the sum of energy consumption for transmitting data is the least in the cluster.

*Prove:* There is *m* nodes distributed in a cluster. Node *i* locates the center of the event area with coordinate (0, 0). From equation (1), energy consumption of communication between nodes is directly proportional to *d^γ^*. Typically, we consider *γ* to be 2. We have

(13) $$D_{i}=\sum_{k=1,k\neq i}^{m}\left(x_{k}^{2}+y_{k}^{2}\right)$$

where *D_i_* is the sum of square distance between node *i* and other nodes in the same cluster. (Node *i* is the CH.)

If node *j* is selected as the CH with coordinate (*x*_j_, *y*_j_), we have

(14) $$D_{j}=\sum_{k=1,k\neq j}^{m}{\left(x_{k}-x_{j}\right)}^{2}+{\left(y_{k}-y_{j}\right)}^{2}$$

We calculate the expectation of (13), (14), respectively, as follows

(15) $$E\left(D_{i}\right)=E\left(\sum_{k=1,k\neq i}^{m}\left(x_{k}^{2}+y_{k}^{2}\right)\right)$$

(16) $$E\left(D_{j}\right)=E\left(\sum_{k=1,k\neq j}^{m}{\left(x_{k}-x_{j}\right)}^{2}+{\left(y_{k}-y_{j}\right)}^{2}\right)$$

According to (15), (16), we have

(17) $$E\left(D_{i}\right)=E\left[\left(x_{j}^{2}+y_{j}^{2}\right)+\sum_{k=1,k\neq \{i,j\}}^{m}\left(x_{k}^{2}+y_{k}^{2}\right)\right]=E\left(x_{j}^{2}+y_{j}^{2}\right)+(m-2)\times E\left(x^{2}+y^{2}\right)$$

(18) $$E\left(D_{j}\right)=E\left[\left(x_{j}^{2}+y_{j}^{2}\right)+\sum_{k=1,k\neq \{i,j\}}^{m}{\left(x_{k}-x_{j}\right)}^{2}+{\left(y_{k}-y_{j}\right)}^{2}\right]$$

where *x* and *y* are the coordinates of a generic node. From (18), we have

(19) $$\begin{array}{l} E\left(D_{j}\right)=E\left[\left(x_{j}^{2}+y_{j}^{2}\right)\right]+E\left[\sum_{k=1,k\neq \{i,j\}}^{m}{\left(x_{k}-x_{j}\right)}^{2}+{\left(y_{k}-y_{j}\right)}^{2}\right]\\ \;\;\;\;\;\;\;\;\;\;\;=E\left[\left(x_{j}^{2}+y_{j}^{2}\right)\right]+(m-2)\times C(x,y) \end{array}$$

*C(x,y)* is given by

(20) $$\begin{array}{l} C(x,y)=E\left({\left(x_{k}-x_{j}\right)}^{2}+{\left(y_{k}-y_{j}\right)}^{2}\right)=E\left(x_{k}^{2}+x_{j}^{2}-2x_{k}x_{j}+y_{k}^{2}+y_{j}^{2}-2y_{k}y_{j}\right)\\ \;\;\;\;\;\;\;\;\;\;\;\;\;\;=2(E\left(x^{2}\right)+E\left(y^{2}\right))-2E^{2}(x)-2E^{2}(y) \end{array}$$

where *E*(*x*) = 0, *E*(*y*) = 0.

From (19), (20), we have

(21) $$\begin{array}{l} E(D_{j})=E\left[\left(x_{j}^{2}+y_{j}^{2}\right)+2\sum_{k=1,k\neq \{i,j\}}^{m}{\left(x_{k}\right)}^{2}+{\left(y_{k}\right)}^{2}\right]\\ \;\;\;\;\;\;\;\;\;\;\;=E\left(x_{j}^{2}+y_{j}^{2}\right)+2(m-2)\times E\left(x^{2}+y^{2}\right) \end{array}$$

From (17), (21), we have

(22) $$E\left(D_{i}\right)<E\left(D_{j}\right),\;\text{if}\;2<m$$

According to the radio model described above, we can infer that the energy consumption is associated with radio distance, *i.e.*, the shorter the radio distance, the smaller of energy consumption would be. Considering (22), we can draw a conclusion that the CH located in the center of event area consumes the least energy for transmitting data.

In order to prolong the network lifetime, we can describe the goal of clustering: maximizing the working time of the cluster, while minimizing the energy consumption in the cluster, which can be formulated as follows:

(23) $$\text{max}\;T_{C},\text{min}\sum_{\left(i,j\in \mathrm{Se}\right)}E_{\mathrm{Se}}$$

where *T_C_* is the working time of a cluster. *E_Se_* is the sum of energy consumption in a cluster.

Since the CH takes on a lot of work in a cluster, the residual energy need be considered when selecting a CH. Based on *Theorem 2* and (23), a node with the higher residual energy, more neighbors and stronger signal strength (*i.e.*, the node is nearer to the signal center) has more opportunity of becoming a CH in the event area. The objective function for becoming a CH, *q_i_*, is given by

(24) $$q_{i}={\left(E_{i}\right)}^{k_{1}}\times {\left(K_{i}\right)}^{k_{2}}\times {\left(SE_{i}\right)}^{k_{3}}$$

where *E_i_* is the residual energy of node *i. K_i_* is a temporary set of node *i*, which is used to store the number of neighbors in the event area. *SE_i_* is the sensed signal strength to an event. *k_1_*, *k_2_*, *k_3_* are parameters to control the weights of *E_i_*, *K_i_* and *SE_i_*, respectively.

Algorithm 1 represents the pseudo-code of cluster formation. *K* is a temporary set which is used to store the number of CH advertisement overheard. There are two timers associated with each sensor: *T_a_* and *T_i_*. *T_a_* is a wait time, when a node located in the event area records the number of neighbors. It is related to network scale. *T_i_* is the waiting time when node *i* broadcast to be a CH, which is given by

(25) $$T_{i}=q/q_{i}$$

where *q* is a coefficient, which is used to control the value of *T_i_*. *T_i_* is inversely proportional to *q_i_*, *i.e.*, the waiting time of a node with the highest *q_i_* is the shortest.

**Algorithm 1:** Cluster Formation

Begin
1: Schedule each node wait time with *T_a_* sec. delay
2: **while** (*T_a_* ≠ 0)
3: **if** Threshold ≤ *RSS_i_***then**
4: **if** node *j* is in the event area and Threshold ≤ *RSS_j_*
5: *K_i_* = *K_i_*+1;
6: **end-if**
7: **end-if**
8: **end-while**
9: **if** Threshold ≤ *RSS_i_***then**
10: node *i* calculates *q_i_* and *T_i_*;
11: **if***T_i_* ≠ 0 **then**
12: wait;
13: collect the sender of any other incoming CH advertisement in *K*;
14: **else**
15: **if***K* = 0 **then**
16: send CH advertisement message;
17: **else**
18: send a join-request to the node *j* (*q_j_* is the biggest);
19: **end-if**
20: **end-if**
21: **end-if**
22: broadcast TDMA schedule to members;
End

Phase I allows only one CH in the event area.

### 4.2. Phase II: Constructing Multipath

In MRP, when the CH needs to deliver data to sink, an improved ACO algorithm is used to establish multiple paths with optimal or suboptimal energy consumption.

There are three kinds of ants in MRP: search ant (SANT), backward ant (BANT) and abnormal ant (AANT). A SANT is used to collect information about multiple paths and intermediate nodes as it travel along the path. A BANT is used to update the pheromone value along the reverse path, and bring information of path to source node, such as residual energy of node, path length and energy consumption of the current path. MRP adds a new type of ant: Abnormal ant (AANT), which is used to partly avoid stagnation of the protocol.

The procedure of searching for multiple paths is as follows:

- The CH creates several SANTs to search for sink. The SANTs gather path information as they travel along the paths.
- The sink creates a BANT when a SANT arrives. The BANT is sent back following the reverse path. When a BANT moves, it need to update the pheromone on the link *(i,j)* at the reverse path.
- When a SANT arrives at an intermediate node, whether or not a AANT is generated according to a small probability.

The following subsections explain the procedure in detail.

#### Search Ant (SANT)

After the cluster formation phase ends, the CH that needs to find several optimal and suboptimal paths to sink sends many SANTs to obtain path information. In order to reduce route discovery time and overheads, the number of SANTs is related to the network scale and the demand of the application.

**Figure 2.**
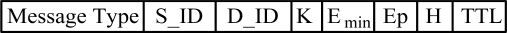
Message Format of a SANT.

The format of message brought by a SANT is shown in Figure 2.

The message type field indicates that it is a SANT. The S_ID field denotes the previous node identification. The field D_ID is next node identification. The K field is the number of a SANT. The E_min_ field gives the minimum energy till the current node. The Ep field gives the sum of energy consumption till the current node. The H field gives the path length so far. The TTL (time-to-live) field gives the depth that a SANT can travel (When a SANT is forwarded, the value of TTL is decreased. That is to say, if TTL reaches zero before the SANT arrives at sink, the SANT message is discarded.).

In order to balance load among nodes and maximize the network lifetime, we modify those equations of the basic ACO as follows:

(26) $$P_{ij}(t)=\{\begin{array}{ll} \frac{\tau_{\mathrm{ij}}^{\alpha }(t)\times \eta_{\mathrm{ij}}^{\beta }(t)}{\sum_{K}\tau_{\mathrm{ij}}^{\alpha }(t)\times \eta_{\mathrm{ij}}^{\beta }(t)}, & \forall j\in N_{i}\;\mathrm{and}\;j\notin M^{K}\\ 0, & \mathrm{otherwise} \end{array}$$

where *P_ij_(t)* is the probability of selecting the next hop node *j* of the current node *i*. *η_ij_* denotes the local heuristic value of the link *(i,j)*, and *τ_ij_* is the pheromone value on link *(i,j)*. *α* and *β* are two parameters used to control the relative weight of pheromone trail and heuristic value, respectively. *M^k^* contains the nodes already visited. In MRP, *M^k^* is kept in the node’s memory instead of keeping in a SANT’s memory. This approach can decrease the size of the data to be transmitted and save energy. *η_ij_* can be given by

(27) $$\eta_{\mathrm{ij}}=\{\begin{array}{ll} \eta_{\text{max}} & \left(\mu_{\mathrm{ij}}>\mu_{\text{max}}\right)\\ \mu_{\mathrm{ij}} & \left(\mu_{\text{min}}\leq \mu_{\mathrm{ij}}\leq \mu_{\text{max}}\right)\\ \eta_{\text{min}} & \left(\mu_{\mathrm{ij}}<\mu_{\text{min}}\right) \end{array}$$

(28) $$\mu_{\mathrm{ij}}=\frac{{\left(E_{j}\right)}^{k_{4}}}{{\left(d_{\mathrm{ij}}\right)}^{k_{5}}}+\theta_{\mathrm{ij}}$$

(29) $$\theta_{\mathrm{ij}}=\{\begin{array}{ll} \frac{{\left(E_{j}\right)}^{k_{4}}}{{\left(d_{\mathrm{ij}}\right)}^{k_{5}}}\times {\left(\frac{d_{\mathrm{is}}}{d_{\mathrm{js}}}\right)}^{k_{6}} & \left(h_{j}\leq h_{i}\right)\\ 0 & \mathrm{otherwise} \end{array}$$

where *k_4_*, *k_5_*, *k_6_* are three parameters, which are used to control the impacts of *E_j_, d_ij_*, *θ_ij_* on *μ_ij_*, respectively. *η*_min_ and *η*_max_ are predetermined parameters.

Equation (30) and (31) are used to update the pheromone value at link (*i*,*j*).

(30) $$\tau_{\mathrm{ij}}(t,t+1)=(1-\rho )\times \tau_{\mathrm{ij}}(t)+\rho \times \Delta \tau_{\mathrm{ij}}^{k}(t,t+1)$$

(31) $$\Delta \tau_{\mathrm{ij}}^{k}(t,t+1)=\lambda \times \left(E_{i}+E_{j}\right)/d_{\mathrm{ij}}^{2}$$

where *ρ* is the evaporation factor, which serves to diminish the intensity of existing trail over time. *λ* is a coefficient. *d_ij_* is the distance between node *i* and node *j. E_i_* is the residual energy of node *i*.

Algorithm 2 represents the basic operations of a SANT. RAND(*x*) is a function to generate a random number uniformly distributed between 0 and *x*.

**Algorithm 2:** A SANT for the Proposed MRP

Begin
1: **if** TTL<>0 **then**
2: **if** a SANT arrives at sink **then**
3: create and release a new BANT;
4: **else**
5: **if***RAND* (*x*)<0.001 **then**
6: create a AANT;
7: the AANT randomly chooses a node as the next hop node;
8: **else**
9: choose the next hop node *j* according to (26)–(29);
10: refresh the residual energy of *i* and *j*;
11: **if** selected node visited **then**
12: back to the previous hop node;
13: re-elect another node as the next hop node;
14: **end-if**
15: using (30), (31) to refresh pheromone value of link *(i,j)*;
16: **end-if**
17 **end-if**
18 **end-if**
End

#### Backward ant (BANT)

When a BANT is back along the reverse path passed by a SANT, the BANT also needs to update the pheromone value on link (*i*,*j*) (Equation (30) and (32) are used to calculate and update the pheromone value.).

(32) $$\Delta \tau_{\mathrm{ij}}^{k}(t,t+1)=c\times \frac{f(t+1)-f_{\mathrm{best}}(t^{*})}{f_{\mathrm{best}}(t^{*})}+c_{1}\times f(t+1)$$

According to (12), we have

(33) $$f(t)=c_{0}\frac{f_{1}{}^{k_{7}}(t)}{f_{2}{}^{k_{8}}(t)f_{3}{}^{k_{9}}(t)}$$

(34) $$f_{1}(t)=\underset{i\in {(n}_{1}{,n}_{2},\cdot \cdot \cdot {,n}_{m})}{\text{min}}\left(E_{i}\right)$$

(35) $$f_{2}(t)=\sum_{(i,j)\subset p}E_{\mathrm{ij}}$$

(36) $$f_{3}(t)=\sum_{(i,j)\subset p}\left(i,j\right)$$

(37) $$f_{\mathrm{best}}(t^{*})=\text{max}[f(t)],t^{*}=1,\cdot \cdot \cdot ,t$$

where *f(t)* is the evaluation function on the current path. *f*_1_*(t)* is the minimum energy node in path *p. f_2_(t)* is the sum of energy consumption in path *p. f*_3_*(t)* is the length of path p. f_best_(t^*^) is the optimal solution so far. (*n_1_*, *n_2_*, …, *n_m_*) denotes the sequence of nodes along path *p. c, c*_1_ and *c_0_* are coefficients, which can be used to control the value of (32) and (33), respectively. *k_7_*, *k_8_* and *k_9_* are weights that determine the relative importance of *f_1_(t)*, *f_2_(t)* and *f_3_(t)*, respectively.

In (32), a scheme of negative feedback is introduced into realizing reward or punishment to the current result. The scheme is helpful of fairness among found multiple paths.

**Figure 3.**
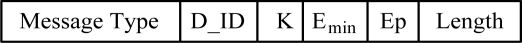
Message Format of a BANT.

The format of message brought by a BANT is shown in Figure 3.

The Message Type field indicates that it is a BANT. The Length field is the path length from sink to the current node. The meaning of other fields is same as that of message brought by a SANT.

Algorithm 3 denotes the basic operations of a BANT.

**Algorithm 3:** A BANT for the Proposed MRP

Begin
1: **if** sink is reached **then**
2: a new BANT is generated;
3: **while** the CH is not reached
4: the BANT moves along the reverse path;
5: the BANT using (30), (32) to update pheromone value of link *(i,j)*;
6: $f_{\mathrm{best}}(t^{*})=\{\begin{array}{ll} f(t) & \mathrm{if}\;f(t)>f_{\mathrm{best}}(t^{*})\\ f(t^{*}) & \text{otherwise} \end{array};$
7: calculate *E_min_*, *E_p_* and *Length*;
8: update the residual energy of *i* and *j*;
9: **end-while**
10: **end-if**
End

### 4.3. Phase III: Data Transmission

MRP is different to these algorithms in [14] because the CH in MRP dynamically chooses one path to transmit data. According to (12), a load balancing function of path *i* is given by

(38) $$f_{i}={\left(E_{\text{min}}(i)\right)}^{k_{10}}+1/{\left(E(i)\right)}^{k_{11}}+1/{\left(\mathrm{Lengt}h_{i}\right)}^{k_{12}}$$

where *k_10_*, *k_11_*, *k_12_* are weight values, *k_10_*+ *k_11_*+ *k_12_* = 1. *E_min_(i)* is the residual energy of the minimum energy node in path *i. E(i)* is the sum of energy consumption in path *i. Length_i_* is the length of path *i*, which can be used to estimate the delay of a path.

(39) $$P_{i}=f_{i}/\sum_{j=1}^{N}f_{j}\;\;\;;\;\;(i=1,\cdot \cdot \cdot ,N)$$

where *N* is the set of discovered paths.

(40) $$\sum_{i=1}^{N}P_{i}=1$$

The CH uses (38)–(40) to calculate the probability and transmit data along the selected path. Since the path is dynamically chosen, load balancing among the paths is achieved.

### 4.4. Route Maintenance

In MRP, route maintenance is responsible for the maintenance of the routes during the communication. The process of route maintenance will be initiated when there comes out these conditions as follows:

- When the residual energy of the current CH is lower than 50% the average energy of all nodes in the cluster, a new CH will be selected according to (24). If there are more than two paths to sink, the new CH will send the packets via these paths. Otherwise, the new CH will initiate a new route discovery process.
- When the number of multiple paths is less than two, that means the reliability of path decreased seriously. The current CH will initiate a new route discovery process.

## 5. Performance Evaluation

Various performance metrics are used for comparing different routing strategies in WSNs. We have used the following:

- *Average Energy:* The metric gives the average of energy of all nodes at the end of simulation.
- *Energy consumption*: The metric gives the energy consumption of nodes in the event area for transmitting a data packet to sink.
- *The standard deviation of energy*: The metric gives the average variance between energy levels on all nodes.
- *Network lifetime*: This metric gives the time of the first node running out of its energy.

By using a simulator developed by MATLAB, the proposed scheme was compared with the TEEN [29] dynamic clustering algorithm and the other two kinds of multipath algorithms in [14] (MP) and [25] (MACS), respectively.

We evaluated these four algorithms over a set of sensor networks with the number of nodes ranging from 100 to 500. For the same number of nodes, we randomly generated ten network topologies and ran these algorithms over them to obtain the average results. In each network, the sensor nodes are randomly distributed on a *M* × *M* region with M = 200 m. For radio power consumption setting, we adopt the first-order model [11] and set *E*_elec_ = 50 nJ/bit, *E*_amp_ = 10 pJ/bit/m^2^. The energy for data aggregation is set to EDA = 5 nJ/bit. The parameters (*k*_1_, *k*_2_, *k*_3_, *k*_4_, *k*_5_, *k*_6_, *k*_7_, *k*_8_, *k*_9_, *k*_10_, *k*_11_, *k*_12_) are set to (0.5, 0.1, 0.4, 2, 1, 1, 0.4, 0.2, 0.4, 0.5, 0.3, 0.2).

Table 2 lists the other simulation settings. *τ_ini_*(*i, j*) is the initial pheromone value at a link (i, j).

$$k=\left\{ \begin{array}{l} 0.01,\text{if}\;\text{node}\;i\;\text{and}\;\text{node}\;j\;\text{are}\;\text{neighbors}\\ 0\;\;\;\;\;\;\;\;\;\;\;\text{otherwise} \end{array}\right..$$

**Figure 4.**
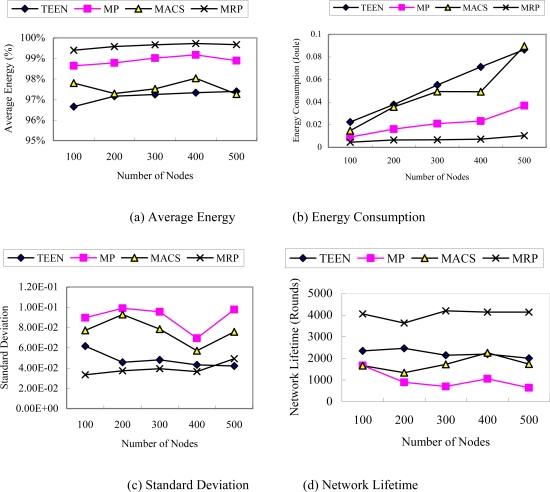
Performance in WSNs with same initial energy levels (scenario 1) (a) Average Energy. (b) Energy Consumption. (c) Standard Deviation. (d) Network Lifetime.

**Figure 5.**
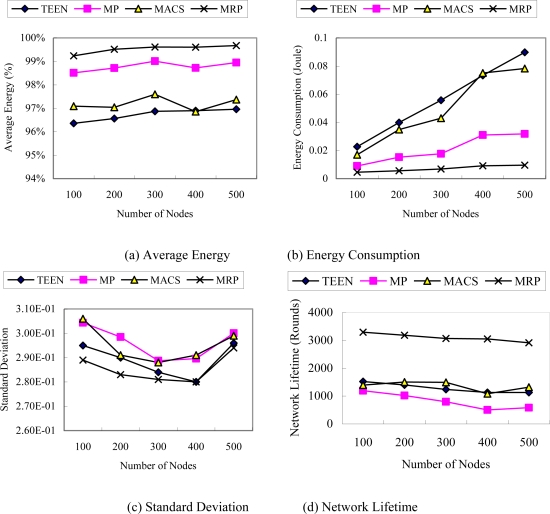
Performance in WSNs with different initial energy levels (scenario 2) (a) Average Energy. (b) Energy Consumption. (c) Standard Deviation. (d) Network Lifetime.

We designed two scenarios to compare the performance of the different algorithms. The first scenario (Figure 4) simulates a homogeneous WSN, where the sensor nodes were randomly deployed with the same initial energy. The initial energy of each node is 2 joules. The second scenario (Figure 5) simulates a heterogeneous WSN where the network is composed of many nodes with different initial energy. The energy level changes between 1 joule and 2 joules, which are uniformly distributed over the nodes.

Figure 4 presents the results of the simulation for the studied metrics to different network scale in scenario one. It can be seen that MRP performed better than the other algorithms.

In Figure 4a, the average energy of the MRP is higher than the other algorithms. This indicates that there exists more residual energy of the nodes in MRP, which implied MRP needs less energy for transmitting data.

Figure 4b shows a linear increase of energy consumption as the network becomes denser, as more sensor nodes become involved for all the algorithms. This brings more traffic into the network. It is obvious that MRP and MP consume less energy than the others. However, MRP outperforms the MP algorithm. Although TEEN also belongs to a dynamically clustering algorithm, the structure of cluster in TEEN is not related to the event area. Therefore, the energy consumption of TEEN is higher than that of MRP. MACS is one of the most costly algorithms, because it fails to take advantage of the data correlation and clustering to remove the redundant information among neighboring nodes. Although MP is also a flat routing algorithm, the energy consumption of MP is less than that of TEEN and MACS. The reason is that the primary route in MP is formed according to some metrics, such as low energy consumption. Therefore, the energy consumption of MP is low when source nodes always use the primary route to transmit data.

In Figure 4c, when compared with the other algorithms, MRP presents a significant reduction in the standard deviation. It indicates that MRP can efficiently balance the energy consumption on all nodes.

Figure 4d shows the network lifetime for the four algorithms. It is evident that the network lifetime of MRP is almost twice of that obtained by the other algorithms. The network size influences MRP the least. The reason is that clustering and dynamically choosing one path to transmit data can greatly contribute to reducing energy consumption and achieving load balancing among all nodes. TEEN outperforms MACS because it can use clustering to transmit data.

Among of the four algorithms, the performance of MP is the worst. The reason is that MP always uses the primary path to transmit data, which results in energy of the nodes in the primary route becoming depleted very soon.

The results illustrated in Figure 5 correspond to the second scenario. From Figure 5, we can see that the results are very similar to those of the first scenario. Although the initial energy of nodes is randomly distributed, MRP still presents the best results. That can be explained by the adaptability of the protocol, which can efficiently balance the energy consumption among nodes by dynamic clustering and using multiple paths to transmit data. The simulation results also illuminate that MRP is suitable for either the homogeneous network or the heterogeneous network.

## 6. Conclusion

For monitoring the burst events in WSNs, we have proposed a novel multipath routing protocol based on clustering and ACO. By introducing an objective function to carry out dynamic clustering, MRP improves the efficiency of data aggregation, thus, reducing the energy consumption. We also use an improved ACO algorithm to search for the optimal and suboptimal paths based on many metrics, which can balance the energy consumption among nodes. Furthermore, a load balancing function is presented for dynamically choosing one path to transmit data. Performance evaluation shows that MRP achieves better load balancing and lower energy consumption, and then, maximizes the network lifetime.

As explained before, MRP has some parameters that need be set. The values of these parameters have a great impact on the performance of the algorithm. For future research, we plan on making the algorithm compatible with different networks by adaptively adjusting the value of these parameters. Furthermore, we intend to extend the algorithm to monitor the object with an appropriate speed.

## Figures and Tables

**Table 1.** Neighbor Information.

**Neighbor ID**	**Pheromone Value**	**Residual Energy**	**Distance to Sink**	**Hop count**	**Tag**
*i*	*τ_ci_*	*E_i_*	*D_is_*	*h_i_*	0
*j*	*τ_cj_*	*E_j_*	*D_js_*	*h_j_*	0
…	…	…	…	…	0

**Table 2.** List of Many Parameters Used.

**Parameter**	**Value**
*α*	2
*β*	2
*ρ*	0.2
*τ_ini_*(*i*, *j*)	*k*
the number of event	1
packet size	512 bytes
broadcast packet size	20 bytes
the coordinate of sink	(0,200)
event radius	20 m
